# Naturally Occurring Mutations in Large Surface Genes Related to Occult Infection of Hepatitis B Virus Genotype C

**DOI:** 10.1371/journal.pone.0054486

**Published:** 2013-01-18

**Authors:** Hong Kim, Seoung-Ae Lee, Dong-Won Kim, Sueng-Hyun Lee, Bum-Joon Kim

**Affiliations:** 1 Department of Microbiology and Immunology, Liver Research Institute, Cancer Research Institute and SNUMRC, College of Medicine, Seoul National University, Seoul, Korea; 2 Department of Microbiology, Family Medicine, and Internal Medicine, Konkuk University school of Medicine, Chungju, Korea; University of Cincinnati College of Medicine, United States of America

## Abstract

Molecular mechanisms related to occult hepatitis B virus (HBV) infection, particularly those based on genotype C infection, have rarely been determined thus far in the ongoing efforts to determine infection mechanisms. Therefore, we aim to elucidate the mutation patterns in the surface open reading frame (S ORF) underlying occult infections of HBV genotype C in the present study. Nested PCRs were applied to 624 HBV surface antigen (HBsAg) negative Korean subjects. Cloning and sequencing of the S ORF gene was applied to 41 occult cases and 40 control chronic carriers. Forty-one (6.6%) of the 624 Korean adults with HBsAg-negative serostatus were found to be positive for DNA according to nested PCR tests. Mutation frequencies in the three regions labeled here as preS1, preS2, and S were significantly higher in the occult subjects compared to the carriers in all cases. A total of two types of deletions, preS1 deletions in the start codon and preS2 deletions as well as nine types of point mutations were significantly implicated in the occult infection cases. Mutations within the “a” determinant region in HBsAg were found more frequently in the occult subjects than in the carriers. Mutations leading to premature termination of S ORF were found in 16 occult subjects (39.0%) but only in one subject from among the carriers (2.5%). In conclusion, our data suggest that preS deletions, the premature termination of S ORF, and “a” determinant mutations are associated with occult infections of HBV genotype C among a HBsAg-negative population. The novel mutation patterns related to occult infection introduced in the present study can help to broaden our understanding of HBV occult infections.

## Introduction

Despite the availability of an effective vaccine, more than 350 million people worldwide are chronically infected with hepatitis B virus (HBV), and many develop serious liver diseases as a result, such as cirrhosis or hepatocellular carcinoma (HCC) [1], [2]. Korea is recognized as an endemic area of HBV infection; based on the Korean National Health and Nutrition Survey of 2007, the prevalence of the HBV surface antigen (HBsAg) was 4.2% in men and 3.1% in women [3]. Moreover, it was reported that the unique epidemiologic trait in this area, an exclusively high prevalence level of genotype C2, which is known to be more virulent than genotype B [4], may contribute to the distribution of characteristic HBV mutation patterns related to the progression of liver diseases [5]–[9].

Occult HBV infection can be defined as the long-lasting persistence of viral genomes of individuals negative for the HBsAg serology [10]–[12]. In general, HBV infection is diagnosed when circulating HBsAg is serologically detected. However, recent progress in molecular-based technology, such as PCR-based methods, has led to the ability to prove HBV infection in HBsAg-negative individuals with or without circulating antibodies to HBsAg and/or the hepatitis B core antigen [13]–[15]. A large body of evidence has shown that HBV occult infection is highly prevalent, particularly in HBV endemic areas, and that it has distinct clinical entities. In particular, HBV occult infection is reportedly related closely to severe forms of liver disease such as cirrhosis and HCC [16], [17]. Furthermore, in patients infected with the hepatitis C virus, HBV co-infection becomes more dangerous [13], [18]–[20].

Although HBV genome sequences from individuals with occult infection have been analyzed, it remains elusive as to which mutations are related to occult infections. Furthermore, genome analyses related to occult infection from genotype C, which is known to be more prone to mutations in the HBV genome [4], have rarely been introduced. Therefore, the aim of the current study is to select mutations related to occult in the surface open reading frame (S ORF) of genotype C and to elucidate the major mechanisms related to occult infection via an analysis of selected mutation patterns.

## Materials and Methods

### Patients and Serum Samples

Serum samples were collected from a total of 624 patients who were negative for HBsAg, 428 from patients visiting Jeju National University Hospital in 2005 and 196 from patients visiting Chungju Hospital of Konkuk University in 2005. All 624 patients were consecutively and randomly selected without consideration of their personal information from the two facilities. In addition, serum samples of 40 patients randomly selected from 120 HBsAg positive carriers visiting Jeju National University Hospital in 2005 were used as the control group. HBsAg, anti-HBs, HBeAg and alanine aminotransferase (ALT) were assayed using a commercial enzyme immunoassay kit (Abbott Laboratory, Wiesbaden, Germany). Carrier HBV DNA was determined quantitatively using a hybrid capture HBV DNA assay kit (Digene, Gaithersburg, MD, USA). Occult HBV DNA levels were measured by means of the Versant HBV DNA 3.0 assay (Bayer Corporation, Tarrytown, NY, USA) [21]. All work was approved by the institutional review board of Seoul National University Hospital (IRB No. C-0803-013-237). The experiment was mainly based on the virion DNA extracted from isolates; therefore the research was done without informed consent and a waiver of informed consent was agreed upon by the IRB. Details of the clinical data are shown in Table 1.

**Table 1:** Comparison of clinical data between the occult subjects and HBV carriers used in this study.

Clinical factors	OccultHBV (41)	Carrier HBV (40)	*P*-value
Age in years, mean ± SD	44.2±18.4	40.1±19.0	NS
Sex, M/F	22/19	21/19	NS
ALT (IU/L) mean (range)	NA	28.4±10.3	NA
HBV-DNA (pg/ml) median (range)	0.01 (0–0.139)*	1812.8 (0–6,000)	NS

NA: not available, NS: not significant.

* Among 41 occult HBV samples, HBV DNA levels from 36 samples were below LOD of the Versant HBV DNA 3.0 assay (<0.007 pg/mL). Because the sensitivity of the nested PCR assay (0.001 pg/mL, data not shown) is higher than the Versant HBV DNA 3.0 assay (0.007 pg/mL), only five from 41 amplified samples were detected.

### HBV DNA Extraction and PCR Amplification

HBV DNA was extracted from 200 <$>\scale 80%\raster="rg1"<$> of obtained serum using the QIAamp DNA Blood Mini Kit (QIAGEN Inc., Hilden, Germany). To elucidate the prevalence of occult infection, we applied the nested PCR protocols while targeting the entire S ORF in all of our 624 Korean HBsAg-negative subjects. Briefly, the first round of PCR was carried out using the sense primer PreS2-Del-F2 (accession number AF286594, positions 2814–2832, 5′ - GGG TCA CCA TAT TCT TGG G - 3′) and the antisense primer HB2R (positions 1029–1049, 5′ - CAT ACT TTC CAA TCA ATA GG - 3′) primers, which target a large surface region, while the second round of amplification was performed using the sense primer Del-PRA-F1 (positions 2887–2907, 5′ - CTT GGG AAC AAG AGC TAC AGC - 3′) and HB2R primers. PCR was initiated using the hot-start technique in a 50 µl PCR mixture containing 2.5 mM MgCl_2_, 400 µM dNTP, and 2.5 U of LA Taq polymerase (Takara, Shiga, Japan). The reaction mixture was subjected to 30 cycles of amplification (60 sec at 95°C, 45 sec at 52°C and 90 sec at 72°C) followed by a 5 min extension at 72°C. A 96-well thermocycler (Model 9600 thermocycler, Perkin-Elmer Cetus, Norwalk, USA) was also used. The obtained PCR products were analyzed by electrophoresis on 1% agarose gels, stained with ethidium bromide, and visualized on a UV transilluminator. This protocol was also applied to the 40 control carriers.

### Cloning and Sequencing Analysis of S ORF

In order to compare the mutation patterns of the preS1, preS2, and S regions between 41 occult subjects and 40 control carriers, the 1378 bp of the PCR products from a total of 81 HBV DNAs were cloned into the TOPO TA cloning kit (Invitrogen Co., Carlsbad, CA, USA). More than four clones per subject underwent a sequencing analysis. Sequencing was conducted using the Applied Biosystems model 377 DNA automatic sequencer (Perkin-Elmer Applied Biosystems, Warrington, UK), which is known to be an automatic sequencing system. As described previously [5], serotypes were determined by a comparison of the amino acid sequences of codons 122 and 160 in the HBsAg of the 41 occult subjects. Mutations were defined through comparisons with 12 reference strains obtained from GenBank [accession numbers M57663 (A), X70185 (A), AB100695 (B), D00329 (B), AB074755 (C1), AF286594 (C2), AY247032 (C2), AY641558 (C2), AY641559 (C2), D16667 (C2), D50519 (C2) and X02496 (D)]. If there were sequence variations between clones of a sample, a typical sequence of variation site was determined as a major sequence. HBV genotypes were determined by a phylogenetic analysis based on eight reference strains representing each of the genotypes of A­D obtained from GenBank [accession numbers M57663 (A), X70185 (A), AB100695 (B), D00329 (B), AB074755 (C1), AY641558 (C2), AB554024 (D) and X02496 (D)]. Among the amplified 1378 bp, only 1203 bp corresponding to coding regions [preS1 (119 aa)+preS2 (55 aa)+S (227 aa) = 401 aa] were subject to the phylogenetic analysis. In the deletions, nucleotides of less than 1203 bp were subjected to an analysis. For a quasispecies distribution analysis of 22 occult subjects with preS deletions (preS1: 15 subjects, preS2: 7 subjects), a total of 93 clones from 22 subjects were subject to a cloning-sequencing analysis. Nucleotides were aligned and their similarities were calculated using the multiple-alignment algorithm in Megalign (DNASTAR, Windows Version 3.12e). Phylogenetic trees were inferred using the neighbor-joining method [22]. Neighbor-joining was carried out using MEGA version 4.0.2 [23]. The resultant neighbor-joining tree and topology were evaluated by bootstrap analyses [24] based on 1,000 re-samplings.

### Statistical Analyses

Results were expressed as percentages, mean SDs, or as medians (range). Differences between categorical variables were analyzed using Fisher’s exact test or a Chi-square test. For continuous variables, the Student’s t-test was used when the data showed a normal distribution, or the Mann-Whitney U test was used when the data was not normally distributed. A p value of <0.05 (two-tailed) was considered to be statistically significant.

## Results

### Prevalence of Occult HBV Infection from Korean Subjects

About 1.3 kbp of PCR amplicons were produced in 41 (6.6%) of 624 samples via the nested PCR assay.

### Genotyping and Serotyping of 41 Subjects with Occult Infections

**Figure 1:**
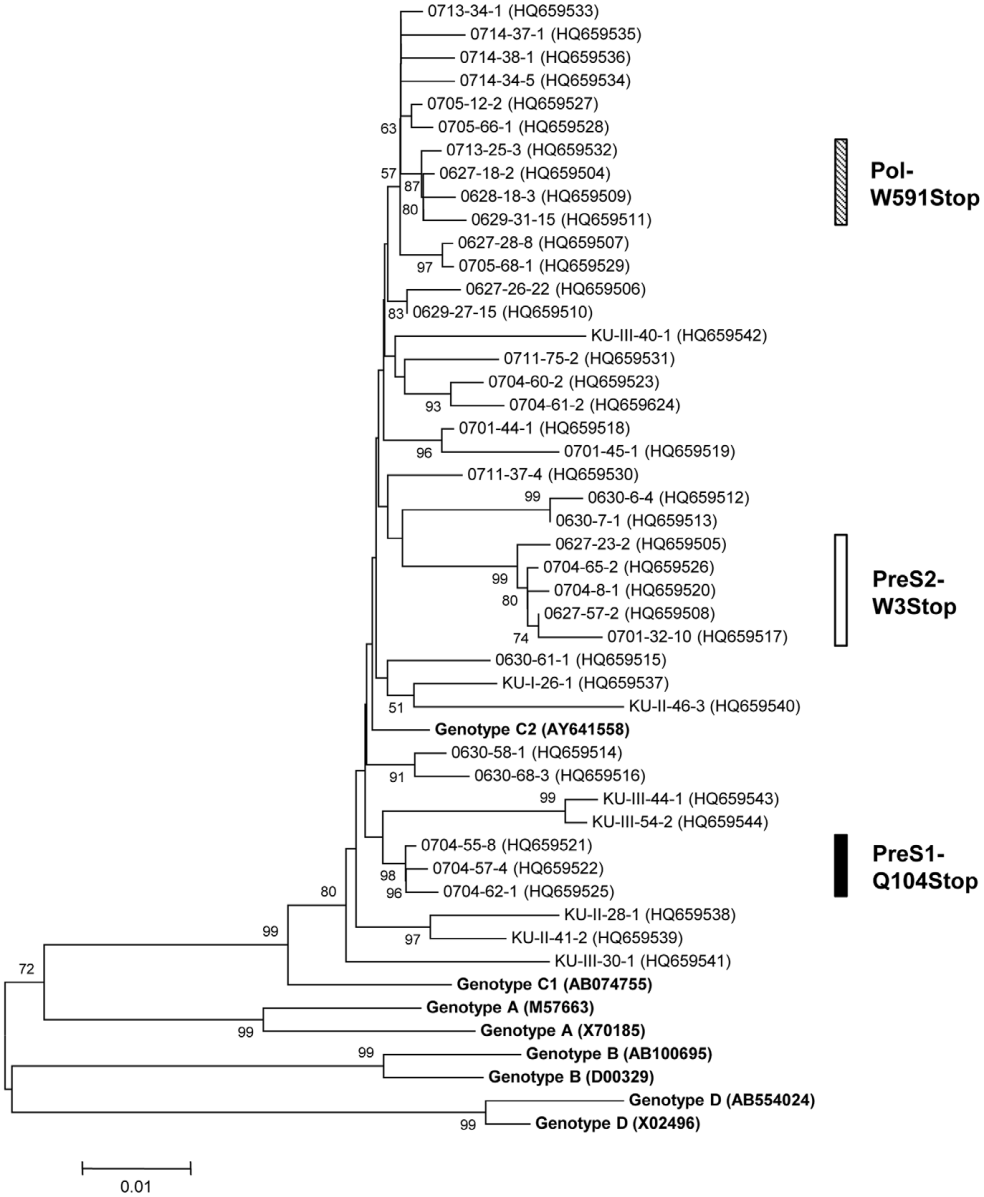
Phylogenetic analysis based on the large surface genome sequences of eight references and 41 HBV occult strains. Genetic distances were estimated using the Kimura two-parameter matrix and the phylogenetic tree was constructed using the neighbor-joining method. The percentages indicated at the nodes represent bootstrap levels supported by 1,000 re-sampled data sets. Bootstrap values of <50% are not shown. The solid circle indicates that the corresponding nodes (groupings) were also recovered in the maximum-parsimony trees. Numbers in parenthesis are Genbank numbers.

The S ORFs of a total of 165 and 113 clones from the 41 occult subjects and 40 carriers were completely sequenced for mutation analyses in the preS1, preS2, and HBsAg gene, respectively. Information on the sequences representing each of the 41 subjects is available in the GenBank database (accession numbers HQ659504–HQ659544). A phylogenetic analysis based on the 1125 bp to 1203 bp sequences showed that all 41 strains from the occult subjects belonged to genotype C2 (Fig. 1). Serotype, *adr*, *adw*, and untypeable were observed in 39 (95.2%), 1 (2.4%), and 1 subjects (2.4%), respectively (data not shown). The untypeable case was due to the presence of an unusual amino acid, serine, at position 160 instead of lysine or arginine [5].

### Sequence Analysis of the Full S ORF of 41 Subjects with Occult Infections

Details of the mutation patterns in subjects with occult infections are presented in Supplementary Table S1. Compared to reference strains, all of the 41 subjects with occult infections always had more than one mutation in their S ORF and an overlapped polymerase (P) region, suggesting an important role of mutation in the S ORF in case of HBV occult infection. Mutations leading to the premature termination of LHBs were found in a total of 16 occult subjects (39.0%) but only one subject in the carriers (2.5%) (39.0% vs. 2.5%, p = 0.001) (Table 2). Premature terminations leading to complete abrogation of normal LHB and MHB production in the preS1 (a change from glutamine to a stop in the 104^th^ codon in preS1, termed PreS1-Q104Stop) and preS2 regions (a change from tryptophan to a stop in the third codon in the preS2, termed PreS2-W3Stop) were found in three occult subjects (0704–55, 0704–57, and 0704–62) and in five occult subjects (0627–23, 0627–57, 0701–32, 0704–8, and 0704–65), respectively. Premature terminations in the HBsAg region were found in a total of eight subjects (0627–18, 0628–18, 0629–31, 0701–44, 0713–25, KU-II-28, KU-II-41, and KU-III-40). Among these, seven were due to the change from tryptophan to a stop in the 182^nd^ codon in the HBsAg (termed sW182*), recently reported to be a hallmark of the progression of liver disease in chronic Korean patients [25]. Of particular interest, four (0627–18, 0628–18, 0629–31, and 0713–25) of the seven subjects with premature termination in the 182^nd^ codon also simultaneously showed premature termination in the 591^st^ codon of the overlapped P region (a change from tryptophan to a stop, termed Pol-W591Stop) (Table S1).

**Table 2:** Comparison of the prevalence of deletion, mutation rates, and substitutions in the preS1, preS2, S and overlapped P regions between the occult subjects and HBV carriers in this study.

Regions	Characteristics		Occult (n = 41) (%)	Carrier (n = 40) (%)	*P*-value
PreS1	Total of Deletion		15 (36.6)	4 (10.0)	0.008
	Premature stop		3 (7.3)	0 (0)	NS
	Mutations	Hepatocyte binding site (aa 21–47)	11 (26.8)	5 (12.5)	NS
		Deletion of Start codon	11 (26.8)	3 (7.5)	0.037
		S17A	5 (12.2)	0 (0)	0.023
		P32L	5 (12.2)	0 (0)	0.023
		W43L/R	6 (14.6)	0 (0)	0.012
		H51P/R	6 (14.6)	1 (2.5)	0.052
		I84T/M	8 (19.5)	1 (2.5)	0.015
	Mutation rates per hundred of amino acids		2.0/100	0.6/100	<0.001
PreS2	Total of Deletion		7 (17.1)	1 (2.5)	0.028
	Premature stop		5 (12.2)	1 (2.5)	NS
	Mutations	W3R/Stop	5 (12.2)	0 (0)	0.012
		S5A	4 (9.8)	0 (0)	0.043
	Mutation rates per hundred of amino acids		1.6/100	0.5/100	<0.001
Small Surface	Premature stop		8 (19.5)	0 (0)	0.003
	Mutations	I/T126N/S	11 (26.8)	3 (7.5)	0.037
		W182L/Stop	15 (36.6)	0 (0)	0.001
	Mutations of the “a” Determinant		15 (36.6)	5 (12.5)	0.019
	Mutation rates per hundred of amino acids		1.4/100	0.6/100	<0.001
Overlapped Polymerase	Total of Deletion		22 (53.7)	5 (12.5)	<0.001
	Premature stop		8 (19.5)	2 (5.0)	0.047
	Mutations in the YMDD region		4 (9.8)	1 (2.5)	NS
	Mutation rates per hundred of amino acids		0.7/100	0.4/100	<0.001
PreS1+ PreS2+ Small Surface	Premature stop		16 (39.0)	1 (2.5)	<0.001

NS: not significant.

### Deletions in preS Region

**Figure 2:**
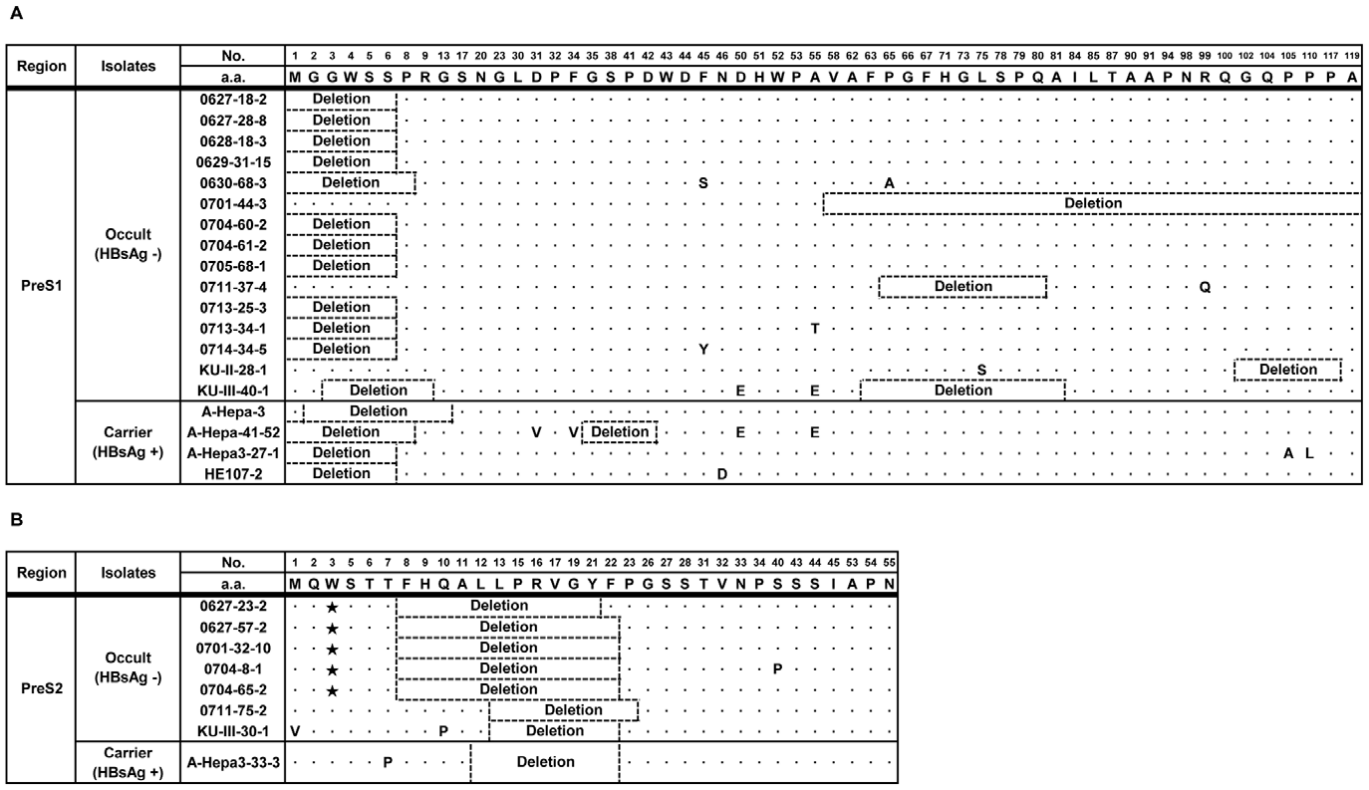
Deletion patterns in PreS1 (A) and PreS2 regions (B) found in occult subjects and carriers. The black star mark indicates the stop codon.

22 (53.7%) of 41 occult subjects had a deletion in the preS1 (15 subjects) or preS2 regions (7 subjects). Notably, no subjects with simultaneous deletions in both the preS1 and preS2 regions were found. The deletion patterns in the preS region of the respective subjects are presented in Fig. 2. The deletion frequencies in preS1 were significantly higher in the occult subjects (15 subjects) than in the carriers (4 subjects) (36.6% vs. 10.0%, p = 0.008). It should be noted that deletion in the preS1 start codon, known to be associated with the progression of liver disease [7], was the most frequently encountered deletion type (11/15 cases, 78.6%) (Fig. 2 and Table 2). The deletion frequencies in preS2 (7 subjects) were also significantly higher in occult subjects than in carriers (1 subjects) (17.1% vs. 2.5%, p = 0.028) (Table 2). All deletions occurred in the region from the 8^th^ to the 23^rd^ codon in preS2, which is identical to the type found in cases of clinical severity in chronic patients [26]. It should also be noted that five of seven occult subjects with the preS2 deletion had simultaneous PreS2-W3Stop mutations leading to premature termination at the preS2 third codon (Fig. 2). A quasispecies analysis of 22 occult subjects with preS deletions indicated that 17 (77.3%) had only deletions without coexistence with wild types. Of note, in seven occult subjects with preS2 deletions, all had only deletions without coexistence with wild types (Table S2).

### Mutation Patterns in PreS Related to Occult Infections

**Figure 3:**
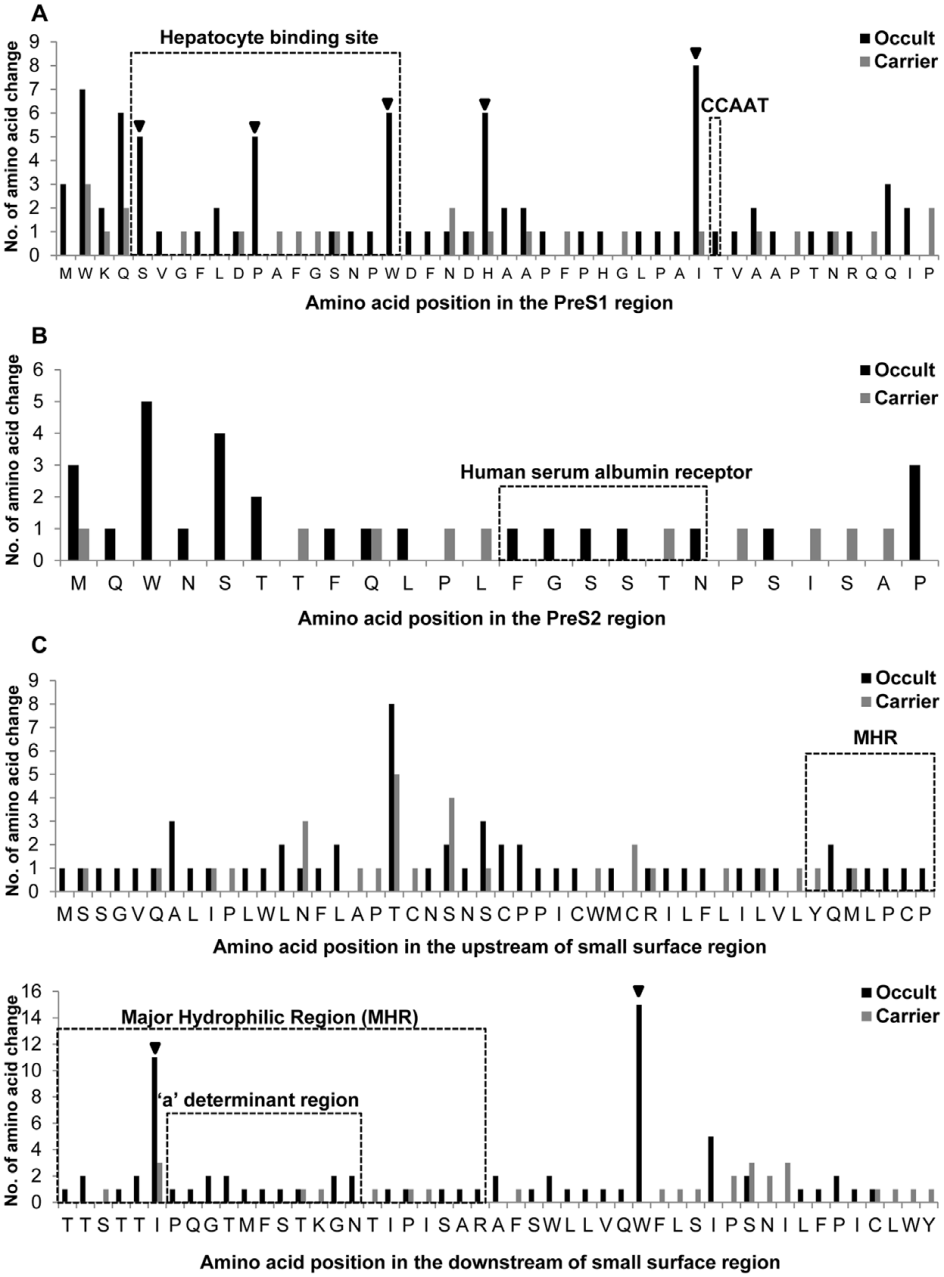
Location of mutated codons in the preS1 (A), preS2 (B) and S region (C) and comparison of the mutation frequencies between occult subjects and carriers. The inverted triangle (▾) indicates codons for which substitutions were significantly or tended to be significantly prevalent in the occult HBV subjects as compared to the carriers.

The mutation frequencies of the preS1 region were significantly higher in subjects with occult infection (2.0%) than in the carriers (0.6%) (p = 0.001). A total of five types of substitution in the preS1 region, i.e., S17A, P32L, W43L/R, H51P/R and I84T/M, each showing higher prevalence in subjects with occult infections than in carriers, were observed (Table 2). Of particular interest, of the five types of substitutions, three types, S17A, P32L, and W43L/R, were located in the hepatocyte binding site in preS1 (Fig. 3), mainly due to the exclusive presence of the PreS2-W3Stop mutation with simultaneous mutations of three types in the hepatocyte binding sites in the occult subjects (Fig. 3, Table S1). Mutation in the CCAAT S promoter region was found only in one subject with occult infection, suggesting it may be not related to occult infection. The mutation frequencies of the preS2 region were also significantly higher in subjects with occult infections (1.6%) than in carriers (0.5%) (p = 0.001). A total of two types of substitution, W3R/Stop and S5A, were found more frequently at statistically significant levels in occult subjects than in carriers. Five of six subjects with premature termination had the PreS2-W3Stop type (Table 2).

### Mutation Patterns in S Related to Occult Infections

The mutation frequencies of the HBsAg region were significantly higher in subjects with occult infections (1.4%) than they were in carriers (0.6%) (p = 0.001). The two types of substitutions in the S region were significantly related to occult infections, i.e., I/T126N/S within the “a” determinant and W182L/Stop outside the major hydrophilic region (MHR). I/T126N/S, reported to be the most frequently encountered among the “a” determinant mutations from chronic Korean patients [9], was found in 11 occult subjects but only in three carriers (26.8% vs. 7.5%, p = 0.037). Instances of W182L/Stop were found in 15 occult subjects but not in the carriers (36.6% vs. 0%, p = 0.001). Two types of mutations in the 182^nd^ codon were found, sW182* and sW182L, in seven and eight subjects, respectively. The sW182Stop mutation was previously reported to be associated with the progression of liver disease of the Korean patients infected with genotype C [25]. Mutations in the “a” determinant were also significantly related to occult infection. They were found in 15 occult subjects but only five in carriers (36.6% vs. 12.5%, p = 0.019) (Table 2).

### Mutation Patterns in Overlapped P Related to Occult Infection

The mutation frequencies of the overlapped P region were significantly higher in subjects with occult infections (0.7%) than in carriers (0.4%) (p = 0.001). Mutations leading to premature termination in the P region were found in eight occult subjects (0627-18, 0628-18, 0629–31, 0704–62, 0711–75, 0713–25, KU-II-41 and KU-III-40) but only in two carriers. A significant difference in the prevalence of mutations pertaining to premature termination was found between the two groups (19.5% vs. 5.0%, p = 0.047). YMDD mutations, reported to be related to lamivudine resistance or HBV replication were also found in four occult subjects, but in only one carrier. Regarding the occult subjects, there was no history of lamivudine use, and it was assumed that patients with YMDD mutations were not given lamivudine as a form of therapy. Clearly, all of the deletions found in the S region can also lead to deletions in the overlapped P region. Therefore, deletions in the P region were found in 22 occult subjects (53.7%, 22/41 subjects) but only in five carriers (12.5%, 5/40 subjects) (p = 0.001) (Table 2).

## Discussion

Because genotype C is known to be responsible for the majority of HBV infections in HBV endemic areas such as China, Korea and other Asia-Pacific nations and given that it is reportedly more virulent than genotype B [4], monitoring of mutation types in genotype C by means of nested PCR, particularly in cases associated with occult infections, should be imperative for the appropriate control of hepatitis B in this area. For example, a specific mutation type in the HBV preS/S region in occult cases may cause an alteration of HBsAg antigenicity or the secretion capacity, which may lead to vaccine escape infections and the emergence of high virulence variants in a population. However, mutations related to occult infection from genotype C isolates have rarely been introduced thus far, despite their probable high frequencies due to the mutation-prone features of genotype C.

A large number of unique mutation patterns in S ORF related to occult infections, thus far not introduced, were in fact found in this study. The high frequency of diverse mutation types related to occult infection in Korean subjects may be due to in part the early introduction of the HBV vaccination among the Korean population. HBV vaccinations were initially introduced in 1983 to the Korean population [27], which dramatically reduced the prevalence of HBsAg-positive chronic carriers from the initial level 30 years ago of more than 10% to 3.7% by 2005 [28]. Selection pressure by this successful vaccination may have led to the generation of diverse mutation patterns capable of causing immune evasion among the vaccinated population [29], [30].

In this study, we investigated mutation patterns related to occult infections of HBV genotype C, focusing on the S ORF from HBsAg-negative Korean subjects. A total of five major characteristic features are noteworthy in terms of mutation patterns related to occult infections from our HBsAg-negative cohort. First, two deletion types in preS1 and preS2, both of which are reported to be associated with liver disease progression in chronic patients, including HCC [7], [8], [31], [32], were significantly implicated in more than half of the occult subjects (53.7%, 22/41 subjects). This finding strongly supports the previous notion that HBV variants related to occult infection can contribute to the progression of HCC or cirrhosis [32]–[34]. Furthermore, HBV mutations in occult infection may be due to de novo infection in patients with advanced liver disease rather than the accumulation of independent mutations in individuals. Our data also showed that these two types of deletions existed in an exclusive manner, suggesting that the simultaneous generation of both deletions may have a lethal effect on the viral life cycle. Of the preS1 deletions, the deletions in the preS1 start codon leading to 11 amino acid truncations of LHBs, as shown in genotype D, were significantly related to occult infection in our cohort. This offers strong support of a previous report showing that a mixed infection of both genotype A and genotype D could lead to HBsAg seronegativity [35]. The preS2 deletion observed in this study may also contribute to occult infection by shortening the distances between the S promoter and the transcription initiation site of S mRNA, in turn leading to an alteration of the expression ratio of LHBs to HBsAg, which can also result in HCC via the up-regulation of the ER stress pathway, affecting the transactivating capacity [31], [32]. The preS2 deletion type is also reported to exist in some occult subjects infected with genotype A or D [36], suggesting its global prevalence in HBV occult infection. Disturbance of the ratios between the three HBV S proteins by LHB overexpression due to these two types of preS deletions may lead to the interruption of virion formation or secretion, thus contributing to HBV occult infection. The questions of whether the two types of preS deletions can lead to the overexpression of LHBs or can contribute to HCC via the ER stress pathway remain to be elucidated in a future study.

Secondly, it is noteworthy that the prevalence of mutations leading to premature termination in LHBs was significantly higher in occult subjects (16 subjects) than in carriers (1 subject) (39.0% vs. 2.5%, p<0.001). The interruption of normal virion formation by this type of mutation may complicate the infection of hepatocytes or the secretion of HBsAg into the blood, leading to occult infection. A phylogenetic analysis of HBV strains showed that three types of premature terminations, PreS1-Q104Stop, PreS2-W3Stop and Pol-W591Stop, all had close relationships within the strains of each type (Fig. 1), suggesting the possibility of horizontal transmission between Korean populations by specific HBV strains related to occult infection.

Thirdly, mutations in the “a” determinant were related to occult infection in our cohort. Mutations in major hydrophilic regions (MHR), particularly the “a” determinant, are known to be associated with occult infection or the generation of vaccine escape variants due to the reduced binding affinity between the HBsAg and the antibody to HBsAg [37]–[40]. Among the MHR mutations related to vaccine escape, the mutation from glycine to arginine in the 145^th^ codon of HBsAg, known as G145R, has been most frequently encountered worldwide [37], [38]. However, the G145R mutation was found only in one patient in our cohort (KU-II-28) (Table S1). The 126^th^ codon of HBsAg was most frequently affected among the MHRs of our occult subjects, thus offering strong support of our previous report which showed that the MHR mutation of the I/T126N/S type prevails in chronic Korean patients with genotype C infection [9]. Of the mutation types in the “a” determinant observed in this study, mutations in two codons, G145 and I/T126 may be related to the global pattern in HBV occult infections.

Fourth, notably, including the W182L and sW182* mutations, the 182^nd^ codon was most frequently affected among the mutations of HBsAg in occult subjects (15/41 subjects). Our previous report showed that HBV variants with sW182* were found at a substantially high frequency in chronic Korean patients of genotype C, particularly in patients with advanced liver disease. Furthermore, a comparison of the clinical data between patients with wild type and sW182* indicated that the HBV DNA levels in patients with sW182* were significantly lower than those with wild types [25], one of the distinctive features of HBV occult infection. This finding provides information about how sW182* can lead to occult infection. However, the exact mechanism of how the sW182* variant leads to occult infection should be addressed in a future study. We also found no W182L mutations in chronic Korean patients of genotype C (data not shown), suggesting that this type of mutation is specific to HBV occult infections. A previous transient transfection study using the HBV full genome construct showed that sW182* interrupts secreted and intracellular virion formation, providing a possible link between sW182* and occult infections [25]. However, the mechanism by which W182L leads to occult infection remains to be found.

We also found that all mutation frequencies with the three aforementioned regions (preS1, preS2 and HBsAg) of S ORF, particularly mutations in the “a” determinant, the major target of the host immune response [35], [39], were significantly higher in occult subjects than in carriers (Table 2), which leads us to speculate that HBV variants related to occult infection may contribute to liver disease progression via persistent infection. Furthermore, our data showing that the preS deletions and sW182* mutations, reportedly related to disease severity in chronic Korean patients, were also found more frequently in occult subjects than in carriers strongly support the above hypothesis. Compared to the mutation patterns in S ORF from occult subjects of other countries, including other genotypes as well as genotype C, two mutation types, deletions in the preS1 start codon and in sW182*, were found to exist exclusively in Korean occult subjects [41].

The most important drawback of our study is that the quasispecies analyses of some samples were based on the very few clones. Therefore, given the low number of templates in these samples, it cannot be excluded that they may be subject to PCR artifact in terms of the accuracy of their mutations.

In conclusion, our data suggest that the defect in virion formation induced by the mutations of preS deletions and sW182* in HBsAg, related to the progression of liver disease in chronic patients, a loss of infectivity via the premature termination of LHBs, and a change in HBsAg antigenicity via “a” determinant mutations, all contribute to the occult infection of HBV genotype C among a HBsAg-negative population. The novel mutation patterns related to occult infection introduced in the present study can help to broaden our understanding of not only HBV occult infections, but also HBV immune evasion.

## Supporting Information

Table S1 **Summary of mutations and deletions in the HBV surface and an overlapped polymerase region observed from 41 occult subjects.**
(XLSX)

Table S2 **Quasispecies analysis of the frequency of the preS deletion from 22 occult subjects with preS deletions.**
(XLSX)
